# Dietary Astaxanthin: A Promising Antioxidant and Anti-Inflammatory Agent for Brain Aging and Adult Neurogenesis

**DOI:** 10.3390/md21120643

**Published:** 2023-12-16

**Authors:** Alessandro Medoro, Sergio Davinelli, Luigi Milella, Bradley J. Willcox, Richard C. Allsopp, Giovanni Scapagnini, Donald Craig Willcox

**Affiliations:** 1 Department of Medicine and Health Sciences “V. Tiberio”, University of Molise, 86100 Campobasso, Italy; alessandro.medoro@unimol.it (A.M.); sergio.davinelli@unimol.it (S.D.); 2 Department of Science, University of Basilicata, V. le Ateneo Lucano 10, 85100 Potenza, Italy; luigi.milella@unibas.it; 3 Center of Biomedical Research Excellence for Translational Research on Aging, Kuakini Medical Center, Honolulu, HI 96817, USA; willcoxbj@gmail.com (B.J.W.); allsopp@hawaii.edu (R.C.A.); d.willcox@okiu.ac.jp (D.C.W.); 4 Department of Geriatric Medicine, John A. Burns School of Medicine, University of Hawaii, Honolulu, HI 96822, USA; 5 Institute for Biogenesis Research, University of Hawaii, Honolulu, HI 96822, USA; 6 Department of Human Welfare, Okinawa International University, Ginowan 901-2211, Japan

**Keywords:** aging, brain, adult neurogenesis, astaxanthin, FOXO3, Nrf2, NF-κB

## Abstract

Decreased adult neurogenesis, or the gradual depletion of neural stem cells in adult neurogenic niches, is considered a hallmark of brain aging. This review provides a comprehensive overview of the intricate relationship between aging, adult neurogenesis, and the potential neuroregenerative properties of astaxanthin, a carotenoid principally extracted from the microalga *Haematococcus pluvialis.* The unique chemical structure of astaxanthin enables it to cross the blood–brain barrier and easily reach the brain, where it may positively influence adult neurogenesis. Astaxanthin can affect molecular pathways involved in the homeostasis, through the activation of FOXO3-related genetic pathways, growth, and regeneration of adult brain neurons, enhancing cell proliferation and the potency of stem cells in neural progenitor cells. Furthermore, astaxanthin appears to modulate neuroinflammation by suppressing the NF-κB pathway, reducing the production of pro-inflammatory cytokines, and limiting neuroinflammation associated with aging and chronic microglial activation. By modulating these pathways, along with its potent antioxidant properties, astaxanthin may contribute to the restoration of a healthy neurogenic microenvironment, thereby preserving the activity of neurogenic niches during both normal and pathological aging.

## 1. Introduction

The brain’s functioning capacities deteriorate with aging. Although this decline varies in the population, it is linked to a reduction in attention, motor coordination, sensory perception, learning, and memory. Likewise, diminished adult neurogenesis, the process by which new neurons are produced and incorporated into the central nervous system (CNS) during adulthood, limited neural plasticity, and neuronal cell death are characteristics of age-related alterations in the CNS [1]. As a result, decreased adult neurogenesis or the gradual depletion of neural stem cells (NSCs) in adult neurogenic niches may occur with aging [2]. Adult neurogenesis plays a key role in CNS remodeling, homeostasis, and neuronal plasticity. Although the existence of adult brain neurogenesis has long been disputed, recent studies have shown that new neurons can be produced in some restricted regions of the brain: the rostral subventricular zone (SVZ) of the lateral ventricles and the subgranular zone (SGZ) of the dentate gyrus (DG) in the hippocampus. Moreover, outside these two main neurogenic sites, although debated, the hypothalamus, substantia nigra, striatum, amygdala, habenula, and cerebellum showed the presence of adult neurogenesis [3].

In the adult brain, a number of factors regulate and control neurogenesis, which has an impact on NSC differentiation and fate. These elements comprise both extrinsic and intrinsic components, including hormones, growth factors, neurotrophins, inflammatory cytokines, physical exercise, and diet [4]. Numerous dietary bioactive compounds have been proven to control and promote neurogenesis in adult progenitor cells. In the adult brain, the growth of NSCs may be enhanced by dietary phytochemicals, which exert many neuroprotective benefits [5,6]. These molecules are able to maintain the particular microenvironments in which adult progenitor cells live beyond their capacity to lower oxidative stress and neuroinflammation and impact growth factors [7,8]. Astaxanthin, a carotenoid mainly extracted from the microalga *Haematococcus pluvialis*, has gained increasing interest in recent years due to its potent antioxidant and anti-inflammatory activities. The main target of this molecule is the brain since, thanks to its unique chemical structure, astaxanthin readily crosses the blood–brain barrier (BBB), thus exerting a protective effect on the human brain by inhibiting neuroinflammation and microglial activation, modulating trophic molecules, attenuating oxidative damage, and activating defensive antioxidant enzymes [9].

This review aims to provide an overview of the current state of knowledge of how astaxanthin may positively influence adult neurogenesis in the context of brain aging.

## 2. Astaxanthin

### 2.1. Sources and Structures of Astaxanthin

Astaxanthin is a reddish-orange xanthophyll, the oxygenated derivative of carotenoids, and is commonly found in the traditional diet of the Japanese island of Okinawa, known for its healthy aging and pro-longevity properties. This diet is rich in astaxanthin due to the high intake of seaweeds, as well as fish and crustaceans [10]. Indeed, the natural sources of astaxanthin are various and include algae, yeast, and marine animals. The green microalgae *H. pluvialis* is one of the primary sources of natural astaxanthin, which is produced as a protective mechanism in response to environmental stress [11]. Salmon, trout, krill, shrimp, and crayfish also contain astaxanthin due to the consumption of astaxanthin-rich organisms like algae or other smaller marine organisms. The astaxanthin content in these sources varies depending on diverse factors such as species, diet, and physiological conditions [12].

Astaxanthin is naturally present in various forms, including stereoisomers, geometric isomers, and both free and esterified forms. Its structure consists of two terminal rings connected by a polyene chain. It has two asymmetric carbons at the 3, 3′ positions of the β-ionone ring, with hydroxyl groups (-OH) at both ends that can be esterified. Indeed, astaxanthin is an unstable molecule that is easily oxidized. Therefore, it is typically found in nature either conjugated with proteins (such as in salmon muscle or lobster exoskeleton) or esterified with one or two fatty acids, forming a mono-ester or a di-ester. Additionally, astaxanthin can exist in different conformations, such as chiral 3S, 3′S and 3R, 3′R, or the uncommon 3R, 3′S form. The main form found in *H. pluvialis* is the 3S, 3′S, which is also predominant in wild Atlantic salmon and occurs mainly in the free-form [13]. The enantiomer 3R, 3′R, mainly in esterified form, is the principal stereoisomer identified in Antarctic krill, *Euphausia superba* [14]. Furthermore, the polyene chain double bond can adopt two distinct conformations: cis or trans. The trans-isomers are more prevalent due to the thermodynamic instability of cis-carotenoids [15].

### 2.2. Safety of Commercial Astaxanthin

Different methods were introduced for astaxanthin extraction, including solvents, acids, edible oils, and microwave-assisted or enzymatic methods [16]. The commercial astaxanthin mainly comes from *Phaffia* yeast, *H. pluvialis*, and through chemical synthesis. Synthetic astaxanthin is a mixture of isomers, including (3S, 3′S), (3R, 3′S), and (3R, 3′R). Astaxanthin extracted from *H. pluvialis* was authorized by the United States Food and Drug Administration (FDA) as a fish feed additive in 1987 and later approved as a dietary supplement in 1999. The European Food Safety Authority (EFSA) has also registered astaxanthin as a fish feed additive, advising an acceptable daily intake (ADI) of 0.034 mg/kg astaxanthin (2.38 mg per day in a 70 kg human) and concluding that the safety of 4 mg astaxanthin per day (0.06 mg/kg) had yet to be fully established [17,18]. However, several studies have indicated that astaxanthin supplementation at doses higher than 4 mg per day has not reported any adverse effects [19,20]. An acute intake of 40 mg astaxanthin was well tolerated by 32 healthy participants, with only three mild events reported within the 48-h following intake [21]. Additionally, a chronic intake of 16 mg and 40 mg per day of astaxanthin has been suggested as safe for patients suffering from functional dyspepsia [22]. These findings suggest that astaxanthin supplementation within the mentioned dosage range is generally well tolerated and safe. Indeed, the astaxanthin extracted from *H. pluvialis* and included in human dietary supplements is safe and accepted by the US FDA at daily doses ranging from 2 to 12 mg and up to 24 mg per day for no more than 30 days [23].

### 2.3. Biological Properties of Astaxanthin

Astaxanthin showed powerful antioxidant properties against reactive oxygen species (ROS) both in vivo and in vitro. The critical role of astaxanthin in redox biochemistry is almost certainly due to its peculiar chemical structure and its exceptional quenching and scavenging abilities. Therefore, it has shown the ability to quench singlet oxygen, scavenge superoxide, hydrogen peroxide, and hydroxyl radicals, and prevent lipid peroxidation [24]. Astaxanthin showed this potential in clinical trials as well. A lysosomal formulation of dark chocolate containing 7 mg of astaxanthin showed interesting effects on the correction of oxidative status in aging individuals with confirmed signs of oxidative stress [25]. In addition to directly interacting with ROS, astaxanthin has been shown to inhibit oxidative damage and inflammation by activating several key genes related to specific biochemical pathways. Indeed, astaxanthin exerts various cytoprotective effects by enhancing the activity of nuclear factor erythroid 2-related factor 2 (Nrf2) and the expression of its downstream antioxidant enzymes [26,27,28,29,30,31]. Under physiological conditions, Kelch-like ECH-associated protein 1 (Keap1) inhibits the activity of the transcription factor Nrf2 by targeting it for ubiquitination and proteasome-dependent degradation. In response to stress, an intricate molecular mechanism facilitates the Nrf2 translocation to the nucleus, where it can promote its antioxidant transcription program [32,33,34]. Numerous studies have additionally shown that the nuclear factor kappa-light-chain enhancer of activated B cells (NF-κB) signaling network, the main pathway involved in the control of inflammatory responses, and the Nrf2 system interact [35]. The ability of Nrf2 to oppose NF-κB has been linked to its involvement in mitigating inflammation. This intricate interplay also involves the role of astaxanthin in the suppression of NF-κB translocation to the nucleus, which in turn suppresses the expression of cytokines in various cellular models [36,37]. However, the beneficial potential of astaxanthin is not limited to its antioxidant and anti-inflammatory abilities; indeed, it showed anti-apoptotic, anti-cancer, and metabolic regulatory properties involving the modulation of numerous intracellular pathways, including those involving the well-known *FOXO3* longevity-associated gene [38,39].

## 3. Adult Neurogenesis and Aging

### 3.1. Mechanisms of Adult Neurogenesis

As a consequence of a process known as adult neurogenesis, the mammalian brain continuously produces new neurons throughout adulthood. However, this phenomenon may not be universal but limited to only specific and constrained brain regions called neurogenic niches, which are unique microenvironments where stem cells are present. The SVZ of the lateral ventricles and the SGZ of the DG in the hippocampus are the two “traditional” neurogenic niches in the adult mammalian brain that have been extensively studied, describing several well-defined stages of the adult neurogenic process [3].

In the adult SVZ, several elements, including growth factors, hormones, and neurotransmitters, may regulate and maintain adult neurogenesis [4]. The type B1 cells, which are the reservoirs of regenerative NSCs, have many characteristics of astrocytes, including several glial markers such as the glial fibrillary acidic protein (GFAP), and differentiate principally in neurons that migrate to the olfactory bulb (OB). Type B1 cells can exist in a quiescent or activated state. Nestin, a marker for undifferentiated neural progenitor cells (NPCs), is expressed when activated type B1 cells undergo asymmetric division, producing type C cells. These type C cells further asymmetrically divide and differentiate into neuroblasts (also called type A cells) [40]. Finally, neuroblasts divide once or more and migrate down the rostral migratory stream, supplying γ-aminobutyric acid (GABA)- and dopamine (DA)-containing interneurons in the OB [41]. As a result, SVZ neurogenesis is crucial for the growth of the olfactory circuitry [40].

The NSCs are also found in the hippocampal DG, towards the edge of the inner granule cell layer, where adulthood-long neuronal renewal is sustained. In the context of learning, memory, and mood regulation, the hippocampus is most likely one of the brain regions with the greatest degree of plasticity. Adult hippocampal neurogenesis may represent an evolutionary mechanism that sustains the functional plasticity of the hippocampus [42]. Hippocampal neurogenesis is, therefore, a complicated, tightly controlled, and multi-step process. Type 1 cells are radial-glia-like cells with nestin and GFAP expression; preserving this pool is crucial for neurogenesis [43,44]. Several factors contribute together to this process, including the transcription factor sex-determining region Y-box 2 (SOX2) and the brain-derived neurotrophic factor (BDNF). In detail, SOX2 modulates several intracellular signaling pathways, which in turn promote and maintain the proliferative state of type 1 cells. In parallel, BDNF activates the tropomyosin receptor kinase B (TrkB) intracellular signaling, activating proteins involved in cell survival and cell migration via protein kinase C [45,46,47]. Asymmetric division of type 1 cells can result in the development of transit-amplifying type 2 cells [48,49]. These transient cells are highly proliferative and can be divided into less differentiated DCX− type 2A cells and more differentiated and committed to the neuronal lineage DCX+ type 2B cells [50,51].

DCX+ type-2B cells undergo additional differentiation to become type-3 cells, which exhibit the neuronal marker polysialylated neuronal cell adhesion molecule (PSA-NCAM) and are nestin-negative. The post-mitotic stage of newly formed cells is characterized by the expression of the neuronal markers neuronal nuclei (NeuN) and calretinin (CR) [52,53]. Only 20% of neuroblasts (immature neurons) survive and are included in the pre-existing neural circuitry [53,54]. After exiting the cell cycle, neuroblasts can establish functional receiving neurotransmitter signals and trophic support, allowing completion of their functional maturation and integration into the preexisting hippocampus circuitry, supporting mood regulation, pattern separation, and spatial and contextual memory [55,56,57]. However, in recent years, new pools of NSCs, induced in response to various pathological and pharmacological stimuli but reduced under physiological conditions, have been reported in several other brain areas with potential neurogenesis activity [58]. These areas include the hypothalamus, which has activities related to energy balance and various homeostatic mechanisms [59,60], the substantia nigra [61], the striatum [62], the amygdala [63], the cortex [64], the habenula [65], and the cerebellum [66].

### 3.2. Aging and Adult Neurogenesis

The effectiveness of the processes responsible for maintaining the homeostasis of the organism and all of its organs and tissues, including the brain and adult neurogenesis, gradually declines with age. Aging, in fact, has a deleterious impact on NSC proliferation, decreasing their capacity to produce new neurons. The reduction in neurogenesis throughout physiological aging brings numerous functional effects, including decreased olfactory discrimination and poorer performance on learning and memory tests. The decline in hippocampal adult neurogenesis in older people is directly connected to a higher risk of memory and cognitive impairment, contributing to neurodegeneration in diseases like Alzheimer’s disease (AD), Parkinson’s disease, frontotemporal dementia, and α-synucleinopathies. However, the presence of specific disease-associated signatures in hippocampal adult neurogenesis revealed its vulnerability to distinct neurodegenerative diseases. This supports the hypothesis that different neurodegenerative diseases may contribute to the imbalance of neurogenic niche homeostasis [67,68,69,70].

Of crucial importance is defining the elements that influence the decline in adult neurogenesis with aging. As previously mentioned, adult neurogenesis needs a certain microenvironment, or “niche”, that offers the signals required to support and regulate the proliferation and differentiation of the precursor cells. During aging, in the neurogenic niche, there is a reduction in the number of actively dividing NSCs and concomitant disruption of the internal homeostatic maintenance, with a consequent marked decrease in precursor cell activity [71]. Numerous intracellular signaling cascades have been considered essential for adult-born neuron survival, including the Wnt/β-catenin pathway. This pathway plays a role in synaptogenesis, pluripotency maintenance, progenitor proliferation, differentiation, migration, and integration into the granule layer. During the aging process, the Wnt pathway is downregulated, which significantly causes the quiescence of NPCs and the consequent reduction in neurogenesis in the hippocampus [72].

Furthermore, the changes that occur with age are known to affect the microenvironmental requirements for maintaining stemness and neurogenesis in niches. These changes in the secretory profiles of nearby niche cells are particularly important. Therefore, as a result of disease or age, alterations in neurogenesis may be caused by changes in growth factor levels. The concomitant microglial increased secretion of proinflammatory cytokines in the SVZ and the age-dependent reduction in the expression of some growth factors in the DG, including insulin-like growth factor-1 (IGF1), fibroblast growth factor-2 (FGF2), and vascular endothelial growth factor (VEGF), may also contribute to the decline in neurogenesis. Indeed, FGF2, IGF-1, and VEGF can individually and positively modulate the proliferation of stem/progenitor cells in the DG; however, their reduced concentrations are linked to dramatically reduced DG neurogenesis in middle-aged mice. Moreover, decreased concentrations of FGF2 during aging are associated with decreased numbers of FGF2+ astrocytes [73,74]. While certain factors may decrease during aging, others, like the cell-cycle regulator transforming growth factor 1 (TGF-1), may become more prominent, causing a reduction in the proliferation of early precursor cells [75]. Additional factors, including increased DNA damage, a lack of proteostasis, and degradation of the vasculature, may limit NSC self-renewal and promote NSC quiescence and death. Blood arteries are, indeed, a crucial and functional component of stem cell niches; the age-related decline in VEGF levels, beyond its negative impact on neurogenesis, may also impair the correct promotion of angiogenesis [76,77].

The numerous cell types of the niches have significantly varied metabolic requirements depending on their current states, and changes in nutrient availability can affect their microenvironment. Nutrient-sensing mechanisms, like the IGF1-forkhead box O (FOXO) pathway, have been shown in numerous studies to be important regulators of NSC function and maintenance of neurogenesis in the SVZ and SGZ. Loss of the insulin/IGF1 pathway’s downstream effectors, the FOXO transcription factors, causes the NSC pool to be prematurely depleted. Likewise, the mammalian target of rapamycin (mTOR) inhibition may improve NSC maintenance because mTOR combines nutrition sensing with biological mechanisms that support cell proliferation [78,79].

Inflammation plays a complicated function in adult neurogenesis, having both positive and negative effects. This dual action can either increase or interfere with neurogenesis and is directly proportional to the intensity of the inflammatory response. It is known that, during acute inflammation, inflammatory mediators typically participate in the homeostasis of neurogenesis as a method of “brain repair”, involving the mobilization of neural precursors for axonal regeneration, remyelination, and repair. On the other hand, uncontrolled high-grade chronic inflammation is a persistent response linked to increased microglia activity with detrimental outcomes for adult neurogenesis. The BBB ordinarily prevents the circulation of proinflammatory mediators in the brain, but with aging, blood vessels become more porous and allow this to happen [80].

During aging, oxidative stress imbalance, such as a surplus of ROS, is one of the major causes of inhibition of adult neurogenesis at different stages, including proliferation, differentiation, migration, integration, and survival of newly produced neurons [81]. According to several studies, persistent oxidative stress significantly reduces neurogenesis in older animals compared to younger animals [82]. Additionally, an increase in ROS production is a key element in activating chronic neuroinflammation and slowing or inhibiting adult neurogenesis [83].

## 4. Astaxanthin and Adult Neurogenesis

### 4.1. Beneficial Effects of Astaxanthin on Molecular Pathways of Adult Neurogenesis

**Figure 1:**
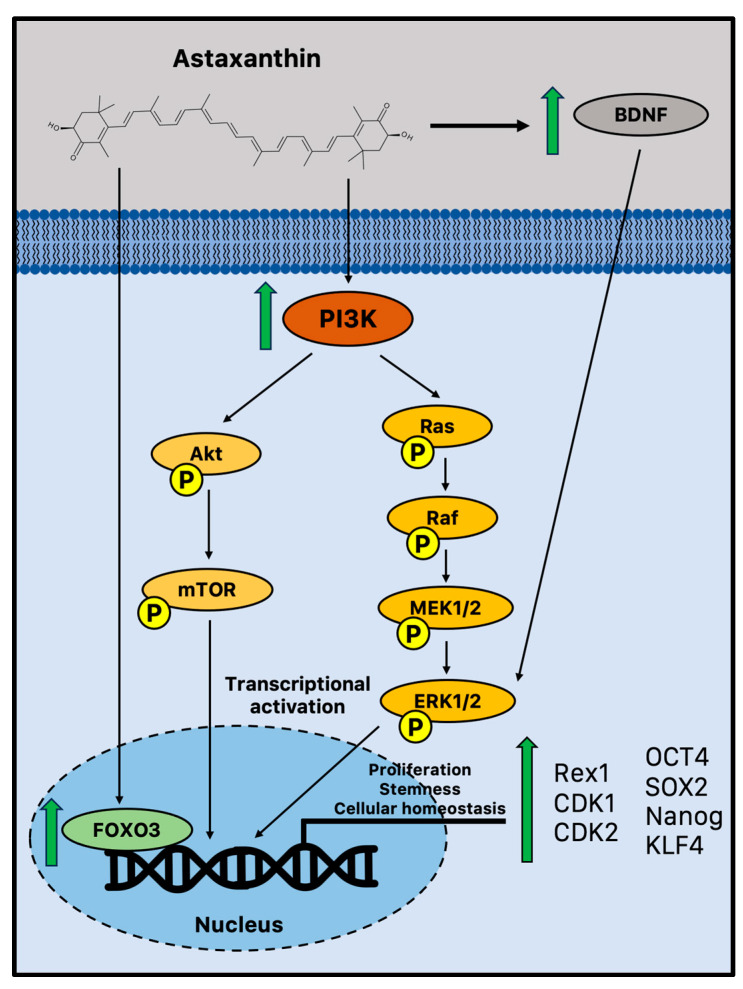
Potential biochemical pathways induced by astaxanthin that sustain adult neurogenesis. Astaxanthin may promote cell proliferation and improve stem cell potency in NPCs via increased activation of FOXO3, PI3K, and MEK signaling pathways [84]. Moreover, in vivo astaxanthin supplementation increased BDNF levels, a fundamental neurotrophic factor in the growth and survival of new neurons via ERK signaling [85,86]. BDNF = brain-derived neurotrophic factor; CDK = cyclin-dependent kinase; FOXO3 = Forkhead box O3; MEK1/2 = mitogen-activated protein kinase 1/2; mTOR = mammalian target of rapamycin; OCT4 = octamer-binding transcription factor 4; PI3K phosphoinositide 3-kinase; Rac = Ras-related C3 botulinum toxin substrate; Raf = rapidly accelerated fibrosarcoma; SOX2 = sex-determining region Y-box 2; and TrkB = tropomyosin receptor kinase B.

There is emerging evidence that suggests astaxanthin may influence neurogenesis and plasticity. It has been reported that astaxanthin can boost the growth of brain precursor cells in vitro (Figure 1).

Indeed, in vitro experiments showed that astaxanthin may improve the NPC potential to proliferate and form colonies in a time- and dose-dependent manner through significant activation of phosphoinositide 3-kinase (PI3K) and its downstream mediators. It upregulated crucial proliferation-related transcription factors, such as Rex1, cyclin-dependent kinase 1 (CDK1), and 2, coupled with the acquisition of active self-renewal activity through the overexpression of canonical stemness genes, including octamer-binding transcription factor 4 (OCT4), SOX2, Nanog, and kruppel-like factor 4 (KLF4) [84]. A study performed on young adult mice (11 weeks) treated for 4 weeks with an astaxanthin-supplemented diet has recently confirmed these in vitro observations on cell proliferation. Therefore, the astaxanthin-enriched food was able to drive cell proliferation in the DG, as evidenced by the increased immunohistochemistry staining of bromodeoxyuridine (BrdU), enhancing hippocampal-dependent cognitive functions [87]. This is not a unique example of astaxanthin-augmented cognitive functions. Indeed, several studies reported improved performances on the Morris water maze, a spatial learning challenge controlled by the hippocampus [88,89,90]. In a recent study, young and old mice were used to assess the impact of astaxanthin on cognitive performance and brain plasticity. In aged mice, one month of astaxanthin supplementation enhanced hippocampal cognitive function and promoted long-term potentiation (LTP), showing intriguing evidence that astaxanthin supplementation can result in functional behavioral results and perceptible cognitive enhancements [91]. Moreover, astaxanthin supplementation ameliorated the behavioral deficits in an AD-like rat model and showed enhanced cognitive and memory abilities in AD models of Wistar rats and ICR mice with anti-senescence and apoptotic effects [92,93,94].

According to a number of reports, astaxanthin is believed to stimulate the pathways that control cell proliferation and differentiation through the influence of extracellular signal-regulated protein kinase (ERK) signaling. ERK and related proteins are significant regulatory molecules that interact with other pathways to integrate signals, leading to diverse endpoints depending on the biological system [84]. This cell signaling cascade is complex as well. This result has some intriguing implications for maintaining cognitive function during aging since ERK signaling plays a crucial role in a variety of hippocampus activities important for learning and memory. Therefore, although ERK has the ability to control other pathways, it is especially required for the generation of LTP in neurons. These subtleties of how astaxanthin regulates ERK signaling as a neuroregenerative or neuroprotective mechanism are still not completely understood. However, the transcription of genes required for synaptic plasticity and neurogenesis can occur when ERK is activated via BDNF, a fundamental neurotrophic factor in the growth and survival of new neurons that declines with aging [85,95]. Astaxanthin supplementation may impact neurogenesis and synaptic plasticity by generating upregulation of BDNF, showing the potential of an effective activator [85]. Moreover, the evolutionarily conserved FOXO family of transcription factors is another intriguing and interesting target of astaxanthin-mediated neuroprotection. Indeed, astaxanthin may influence adult neurogenesis by activating FOXO3, a transcription factor that may directly influence the genetic network that sustains a healthy pool of mammalian stem cells for lifelong neurogenesis [96].

### 4.2. Positive Effects of Astaxanthin on Molecular Pathways of Neuroinflammation and Oxidative Stress

The aging brain with impaired neurogenesis exhibits increased oxidative stress and inflammation, which are also pathogenic traits of many neurodegenerative disorders, including AD. Notably, the chronic activation of microglia is a significant source of reactive oxygen species (ROS) in the brain during chronic inflammation. A substantial effect of astaxanthin is related to dual microglia regulation by preventing enhanced oxidation and pro-inflammatory activation through different molecular mechanisms [9].

#### 4.2.1. Oxidative Stress and Nrf2 Pathway

**Figure 2:**
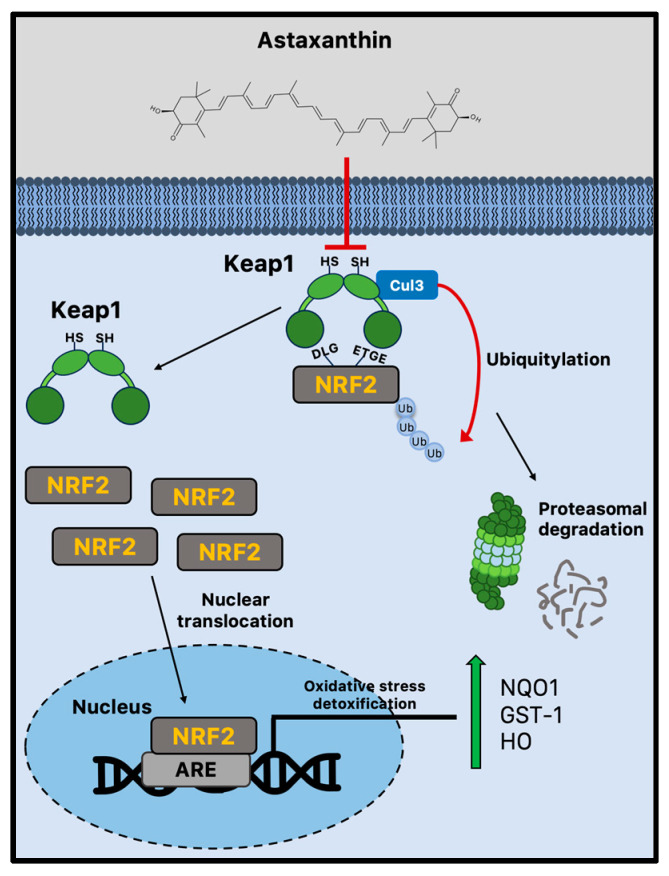
Nrf2-mediated antioxidant mechanism activated by astaxanthin during brain aging. Astaxanthin may decrease Nrf2 proteasomal degradation via the Keap1 pathway. Astaxanthin stimulates Nrf2 nuclear translocation and transcriptional activation of antioxidant genes through the ARE sequences. ARE = antioxidant responsive element; Cul3 = cullin3; GST-1 = glutathione-S-transferase 1; Keap1 = Kelch-like ECH-associated protein 1; HO-1 = heme oxygenase 1; Nrf2 = nuclear factor erythroid 2-related factor 2; and NQO1 = NADPH quinone dehydrogenase 1.

Due to cellular senescence and aging, the abnormal accumulation of ROS can affect brain cellular components, impairing adult neurogenesis [82]. Astaxanthin shows stronger antioxidant activity compared to other carotenoids, including lycopene, α-carotene, β-carotene, and lutein [13]. For example, compared to α-tocopherol, it can neutralize singlet oxygen (^1^O_2_) around 550 times more effectively [97]. Both the polyene system present in other carotenoids and the terminal rings that are specific to astaxanthin’s structure contribute to its potent antioxidant potential [98]. Astaxanthin can donate an electron, create chemical bonds with reactive species, and absorb free radicals into the polyene chain. Unsurprisingly, there is a plethora of experimental evidence showing that astaxanthin can lower ROS in vitro [99], and more subsequent studies have confirmed these early findings in animal models [100,101,102]. The administration of astaxanthin is regularly linked to decreased oxidative damage markers [99,101,103], although its mechanisms of action go well beyond its capacity to scavenge free radicals. Indeed, strong evidence suggests that astaxanthin may boost endogenous antioxidant enzyme activity [104]. Nrf2 is a crucial transcription factor that protects redox homeostasis and is thought to be a potential therapeutic target for diseases linked to oxidative stress and inflammation [105,106]. Numerous cytoprotective and antioxidant genes, including NADPH quinone dehydrogenase 1 (NQO1), glutathione-S-transferase 1 (GST-1), and heme oxygenase 1 (HO-1), are activated by Nrf2 [107]. By enhancing the activity of the PI3K/Akt and ERK pathways, astaxanthin seems to activate and upregulate the Nrf2 pathway [26,27]. Multiple studies produced conflicting results, making it unclear how astaxanthin influences Keap1 activity [28,29]. Indeed, astaxanthin seems to decrease Nrf2 degradation, disrupting the interaction with Keap1 [30]. However, the disruption of the Keap1/Nrf2 complex is not dependent on ERK activation, even though astaxanthin can boost ERK activity, indicating that astaxanthin stimulates Nrf2 nuclear translocation via a different mechanism (Figure 2) [26].

By reducing oxidative stress, astaxanthin reduces neuroinflammation and may have a positive neuroprotective effect on neurogenesis. Therefore, it has been shown that astaxanthin can prevent apoptosis by directly protecting NPCs from oxidative insults, such as exposure to 0.3 mM H_2_O_2_, in addition to promoting cell replication [108]. Moreover, astaxanthin administration protected against neuronal loss in the hippocampus of an epilepsy rat model and prevented oxidative stress, neuroinflammation, and a reduction in Nrf2 levels, alleviating epilepsy-induced cognitive impairment in another model of rats [36,101]. Similarly, astaxanthin reduced oxidative stress and neuroinflammation via the PI3K/Akt/Nrf2 pathway to protect against lanthanum oxide nanoparticle-induced hippocampus damage [109]. Furthermore, astaxanthin may reduce oxidative stress, neuroinflammation, and memory impairment induced by lipopolysaccharide (LPS) and protect against doxorubicin-induced memory impairment by preventing oxidative, inflammatory, and pro-apoptotic insults [110,111].

#### 4.2.2. Neuroinflammation and NF-κB Pathways

One characteristic of chronic neuroinflammation is the abnormal synthesis of pro-inflammatory cytokines in the CNS. The resident macrophages in the brain, which are known as microglia, respond to cellular insults by generating pro-inflammatory cytokines, including interleukin 1 (IL-1), IL-6, tumor necrosis factor (TNF), and nitric oxide (NO). During aging, chronically higher amounts of cytotoxic pro-inflammatory compounds are produced as a result of age-related chronic microglial activation, which damages healthy brain tissue. Indeed, “primed” microglia not only develop hyperreactivity, producing inflammatory mediators even in the absence of an immunological stimulus, but they also lose sensitivity to the signals that regulate microglia activation. Age-related increases in microglial activation and inflammation are known to be associated with diminished neurogenesis and, consequently, cognitive performance [112]. By suppressing the NF-κB pathway, the levels of several pro-inflammatory cytokines, such as TNF, IL-1, and IL-6, can be reduced by the action of astaxanthin, helping to reduce neuroinflammation and chronic microglial activation and restore the appropriate microenvironment in the neurogenic niches [113].

For microglial immunological responses, the NF-κB transcription factor family is essential. This family in mammals is formed by p65 (RelA), RelB, c-Rel, p105/p50 (NF-κB1), and p100/p52 (NF-κB2). These proteins form homo- and heterodimeric complexes that are transcriptionally active. Under normal conditions, the interaction between these NF-κB dimers and IκB proteins keeps them in an inactive form within the cytosol. The IκB kinase (IKK) complex phosphorylates these inhibitors, which causes NF-κB translocation to the nucleus and activation of target gene transcription [114].

**Figure 3:**
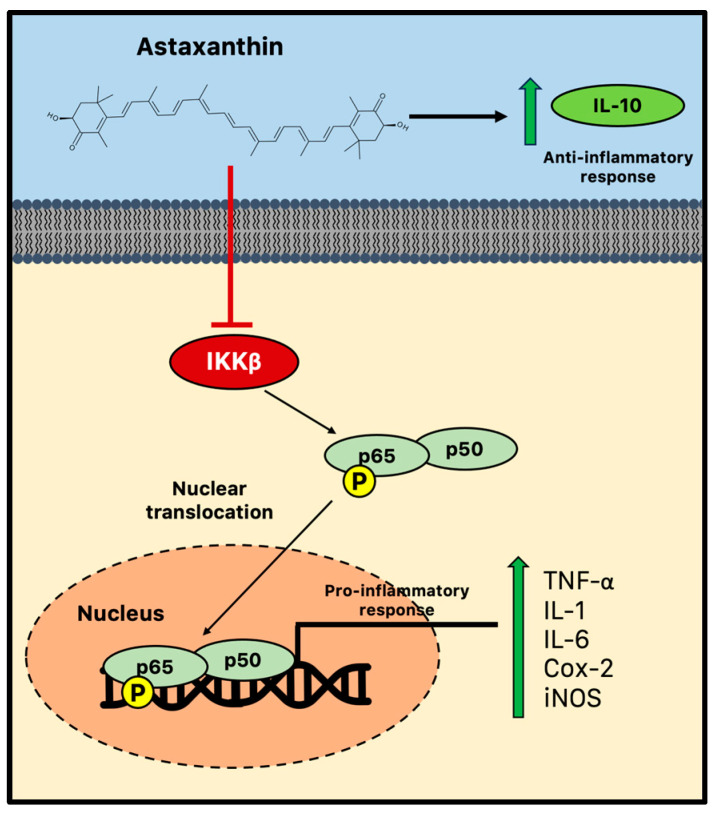
Anti-inflammatory activity of astaxanthin during chronic neuroinflammation. Astaxanthin may effectively reduce microglial-induced neuroinflammation by suppressing the NF-κB pathway and impairing the consequent nuclear translocation and the DNA binding activity of p50/p65 dimers. Cox-2 = cytochrome c oxidase subunit II; IKK = IκB kinase; IL = interleukin; iNOS = inducible nitric oxide synthase; and TNF = tumor necrosis factor.

There is growing evidence that astaxanthin prevents neuroinflammation by selectively modifying microglial activity and blocking the canonical NF-κB pathway. Astaxanthin, for instance, blocked the induction of NF-κB by several inflammatory factors, such as cytochrome c oxidase subunit II (Cox-2), IL-1, and TNF in the hippocampus and parahippocampal cortex, in a rat model of status epilepticus [36]. In parallel, in vitro astaxanthin treatment was able to decrease the expression and release of IL-6, Cox-2, and inducible nitric oxide synthase (iNos)/NO via NF-κB-mediated signals in microglial cells stimulated with LPS [37,115]. Additionally, by controlling the NF-κB pathway, astaxanthin successfully counteracted LPS-induced TNF-α, IL-1, and IL-6 production in the hippocampus and prefrontal cortex [116]. A high dose of astaxanthin (75 mg/kg) was also found to dramatically reduce NF-κB DNA binding activity, inflammatory cytokine production, and intercellular adhesion molecules following subarachnoid hemorrhage in rats. Therefore, astaxanthin can effectively reduce NF-κB-induced inflammation by suppressing the phosphorylation mediated by IKKβ, a crucial event of the cytokine-activated intracellular signaling pathway involved in triggering immune responses and the nuclear translocation of p65 [117]. Furthermore, astaxanthin may impair the nuclear translocation and the consequent DNA binding activity of p50/p65 dimers through the promotion of the phosphorylation of p65 (Figure 3) [118].

Decreased NF-κB and neurodegeneration in the frontal cortex and hippocampus were related to enhanced performance in the Morris water maze in astaxanthin-treated mice. Dietary supplementation with astaxanthin was able to reduce the expression of a number of transcripts linked to inflammation. Aging is known to increase pro-inflammatory markers such as complement component 1q chain A (Cq1A), glial acidic fibrillary protein (GFAP), and cathepsin, an enzyme that helps deliver MHC II antigens. The decrease in these age-related pro-inflammatory markers raises the possibility that astaxanthin has the ability to slow the transition to a pro-inflammatory microenvironment, which is frequent during aging and associated with impaired neurogenesis [119]. Moreover, oral administration of astaxanthin (10 mg/kg) for 6 weeks was enough to modify the expression of specific cytokines in old rat brains. However, the cytokine levels across the different brain regions were found to vary by gender. For example, in the hippocampus and cerebellum of old female rats, it was found that astaxanthin supplementation decreased pro-inflammatory IL-1. Contrarily, the immunomodulatory IL-10 levels increased in the male hippocampus while decreasing in the female cerebellum. Considering that IL-10 can block NF-κB activity, suppressing cytokine secretion in microglia, the ability of astaxanthin to augment the production of anti-inflammatory mediators like IL-10 is crucial for the restoration of trophic and repair functions of microglia and the normal microenvironment of neurogenic niches, which are altered in the context of aging [120].

## 5. Conclusions

In recent years, many findings have shown that astaxanthin has beneficial effects on human health beyond its antioxidant properties. Its unique chemical structure, which is responsible for its antioxidant potential, also enables it to cross the BBB and interact with various molecular pathways, representing a non-pharmacological approach to slowing down the effects of aging on adult neurogenesis. Recent evidence has demonstrated that astaxanthin can promote molecular pathways involved in adult neurogenesis, enhancing cell proliferation and the potency of stem cells both in vitro and in animal models. Additionally, its anti-inflammatory properties may help limit neuroinflammation that is associated with aging and chronic microglial activation. By modulating these pathways, along with its antioxidant properties and the regulation of neurotrophic factors, astaxanthin may help preserve the activity of neurogenic niches during both normal and pathological aging, as largely demonstrated in animal models, ultimately helping to maintain cognitive functions in the elderly. However, while these findings are promising, the understanding of the molecular signaling cascades by which astaxanthin may improve adult neurogenesis is very limited. Moreover, the clinical application of astaxanthin is still largely undetermined due to the difficulty of verifying its molecular and cellular effects in clinical trials. These studies may help to assess the potential of dietary astaxanthin in improving the aging brain and the optimal daily dose for these beneficial effects.

## Data Availability

The original data presented in the study are included in the article; further inquiries can be directed to the corresponding author.
